# An attempt to distinguish physical and socio-psychological influences on pedestrian bottleneck

**DOI:** 10.1098/rsos.211822

**Published:** 2022-06-01

**Authors:** Jonas Rzezonka, Mohcine Chraibi, Armin Seyfried, Ben Hein, Andreas Schadschneider

**Affiliations:** ^1^ Institute for Advanced Simulation, Forschungszentrum Jülich GmbH, Julich, Germany; ^2^ School of Architecture and Civil Engineering, University of Wuppertal, Wuppertal, Germany; ^3^ Institute for Theoretical Physics, University of Cologne, Cologne, Germany

**Keywords:** crowds, bottleneck, social psychology, physics, simulation, pedestrian

## Abstract

It has been realized that the distinction between social-psychological effects and physical effects in pedestrian crowds is complex, and so the relevance of social psychology for the properties of pedestrian streams is still discussed controversially. Although physics-based models appear to capture many properties rather accurately, it was argued that simple systems of self-driven particles could not explain certain emergent phenomena. In particular, results from a recent empirical study of pedestrian flow at bottlenecks have been interpreted as indicating the relevance of social psychology even in relatively simple scenarios of crowd dynamics. The study showed a surprising dependence of the density near the bottleneck on the width of the corridor leading to it. The density increased with increasing corridor width, although a wider corridor provides more space for pedestrians. It has been argued that this observation is a consequence of social norms, which trigger the effect by a preference for queuing in such situations. However, convincing evidence for this hypothesis is still missing. Here, we reconsider this scenario from a physics perspective using computer simulations of a simple microscopic velocity-based model.

## 1. Introduction

Arguably the most important scenario in crowd motion is bottlenecks, i.e. local restrictions of the flow. Such bottlenecks appear in different situations, e.g. people entering a lecture hall or evacuations from large rooms or buildings. Due to their importance, several experimental studies on bottlenecks have been performed during the last two decades [1–13] in an effort to understand the relevance of various factors on the flow properties of pedestrian streams. The results show that a multitude of factors like the geometry of the bottleneck, its width, the number of pedestrians and their motivational mode have a significant impact on the flow and density. A comprehensive summary of the different effects found empirically in bottleneck flow was recently published and discussed the controversy between different experimental results [11].

What is not well understood up to now is the role of human psychology in such (relatively) simple scenarios. It could be argued that this is different for emergency situations and crowd disasters, where the occurrence of casualties and injuries is often attributed to ‘panicking’ pedestrians [14,15]. However, this interpretation has been thoroughly challenged by many studies showing clearly that behaviour colloquially associated with the notion of panic, e.g. irrational and competitive behaviour that leads to the trampling of falling pedestrians [14,16], is actually almost never observed [16].

Besides empirical studies, many investigations use computer simulations based on a wide range of modelling approaches [17–27]. These provide the means to investigate microscopic interactions governing the dynamics of crowds in a wide range of situations, from normal modes [25] to high motivation egress and emergency evacuations [18,19].

More detailed studies of bottlenecks have revealed a number of interesting self-organization phenomena. In 2005, Hoogendoorn and Daamen experimentally observed a boundary-induced formation of lanes inside a spatially extended bottleneck [1] where the pedestrians walk side by side in different ‘lanes’. In narrow bottlenecks, where due to the shoulder width two separate lanes would be wider than the bottleneck, a zipper effect occurs. In this case, two lanes can overlap where pedestrians of one lane partially occupy the longitudinal gap between two successive pedestrians in the neighbouring lanes, as in a zipper. With increasing width of the bottleneck, the lateral distance between lanes increases, whereas the longitudinal gap becomes smaller. This allows for a denser configuration of pedestrians and leads to a linear increase in the capacity for such zipper-like intertwined lanes instead of a stepwise increase that would be expected for non-overlapping lanes [2].

**Figure 1.**
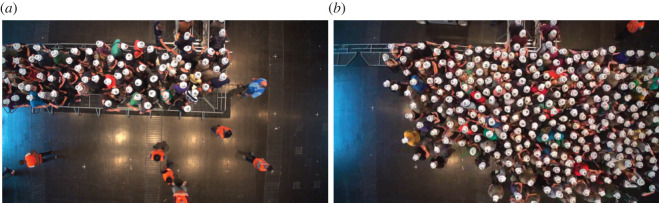
Photos of the experiment modified from [3]. (*a*) The corridor set-up. (*b*) The unrestricted set-up.

The aim of this study is to illustrate the difficulty in investigating and distinguishing social psychological phenomena affecting the behaviour of pedestrians from physical effects. This can lead to the assertion of social psychological influences, where physical effects are sufficient to explain the phenomena. We illustrate this for a specific example of pedestrians’ behaviour at bottlenecks based on two recent experimental studies [3,4]. Experiments performed in 2013 [3] aimed to understand the relevance of social norms, specifically which rules of conduct apply and when these rules are addressed. The study compared the behaviour and stream properties for two different spatial structures of guidance barriers near an entrance that act as bottlenecks. The two set-ups were a semicircle and a corridor scenario with guiding barriers from the side in front of two entrances (figure 1) as is often used by crowd managers. In both cases, the participants were distributed loosely to ensure low initial densities. As motivation, the participants were told that the first to pass through the entrance would ensure a good place in front of a stage in a concert hall. Surprisingly, it was found that the densities in front of the entrance were higher in the semicircle set-up. The corridor scenario results in significantly lower densities and motion in ordered lanes with almost no pushing. By contrast, in the semicircle set-up participants tried to fill empty spaces quickly, which sometimes resulted in pushing others. A possible interpretation of these observations is that the physical boundary conditions influenced the participants’ social behaviour. In order to elucidate this further and to exclude other potential influences, only corridor width and motivation were varied. These experiments were performed in a straight corridor of variable width (between 1.2 m and 5.6 m) to reduce the influence of the geometry. Furthermore, runs with different motivations (high and low) were performed. For the high motivation case, the participants were told that only a restricted number of front row positions with a good view in a concert hall are available.

Two major effects were observed. For a given motivational mode, the density in front of the bottleneck increases with the corridor width. Changing from low to high motivation, the density increases for all corridor widths (figure 5*a*). To gain more insight into the influence of social norms, the participants were asked to fill out a questionnaire. The analysis casts doubt on the initial claim that the dynamics cannot be explained without social psychology, as social behaviour like queuing norms could not be detected. By contrast, a theoretical study based on simulations with a cellular automata model [18] found some indications for an increasing density. However, due to the discrete nature of cellular automata and the resulting coarse (spatial) resolution, the microscopic interactions and emergent phenomena could not be studied in sufficient detail. Therefore, in this study, we will use a different model to investigate the experiment by Adrian *et al.* [4] theoretically. Our approach is based on a velocity-based model with rather simple interactions between the agents. Furthermore, we allow only for a minimal variance in the parameters modelling the individual behaviour.

On a technical side, the main goal is to investigate whether the experimental observation, the increase of the density with the width of the corridor, can be reproduced with a model not requiring complex inner functions for the agents’ behaviour. Also, it is investigated whether another experimental observation, the change of the density with the motivation, can be reproduced by adapting a model parameter that describes how pedestrians close gaps that arise. The general aim is not to diminish the possible influences of social psychology on crowd behaviour, but to highlight the difficulty of distinguishing social psychological influences from physical effects and the need for a framework that adequately addresses this problem.

## 2. Experimental data

**Figure 2.**
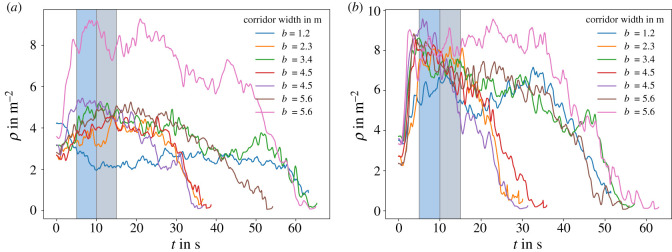
Time series of the Voronoi density from the experiments [4] in the straight corridor for low motivation (*a*) and high motivation (*b*). The blue and grey areas mark the region between 5 and 10 s and 10 and 15 s. The measurement of the density is conducted in the area according to figure 3*b*.

**Figure 3.**
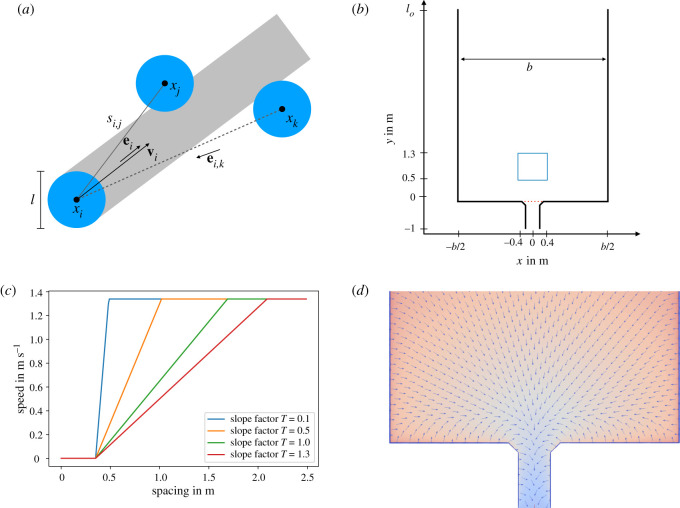
(*a*) Illustration of the collision-free speed model. Agents that overlap with the grey area are in the set *J*_*i*_. (*b*) Illustration of the geometry for all scenarios. The blue square is used for density measurement, the red dashed line for flow measurement. (*c*) Illustration of the speed function (3.2). (*d*) Illustration of the floor field with wall avoidance. The colour illustrates the time cost *c*(**x**) and the vectors indicate the direction of ∇c(x) in (3.5).

The data [4,28] from the experiments conducted in a straight corridor are reanalysed in this study, to allow for a better comparison with the simulations. A summary of the experimental conditions in each run is given in table 1. The geometry of the corridor is illustrated in figure 3*b*. Measurements for the mean density are made in a rectangular area between x={−0.4,0.4} m and y={0.5,1.3} m. The outflow and exit times are measured at the *y* = 0 m line. The methodology for determining the mean density is described in the section ‘Material and methods’. The mean density time series for the different experimental runs are shown in figure 2 both for the experiments with low and high motivation. These are all runs considered in this study. Runs with fewer than 42 participants (i.e. 20–25 participants) have been discarded here since it was not clear whether a consistent steady state has been reached. In the experimental study, the measurement time interval for the mean density is from 5 s to 10 s to have comparable conditions, considering the varying and limited number of participants. In this study also the interval between 10 s and 15 s is measured to be closer to a steady state as can be observed for most runs in figure 2. Only the runs for *b* = 4.5 m in the high motivational case (figure 2*b*) do not reach a consistent steady state as the mean density quickly peaks between 5 s and 10 s and decreases from there on. The mean values for the experiments in the 5–10 s and 10 –15 s interval are shown in figure 5*a*.

**Table 1.** Summary of the experimental conditions in [4] relevant for this study. Run is an identifier for the specific run of the experiment with corridor width *b*, the motivation being high (hi) or low (lo) respectively and the number of participants *N*.

run	width *b* in m	motivation	*N*
030/040	5.6	hi/lo	75
050/060	4.5	hi/lo	42
070/080	2.3	hi/lo	20
090/100	1.2	hi/lo	24
110/120	1.2	hi/lo	63
150/160	5.6	hi/lo	57
170/180	1.2	hi/lo	25
190/200	3.4	hi/lo	22
230/240	2.3	hi/lo	42
200/260	4.5	hi/lo	42
270/280	3.4	hi/lo	67

## 3. Model

The simulations use a velocity-based model [17] with simple agent interactions and a noise term in the desired direction. The interactions are similar to volume exclusion in cellular automata models, while the desired direction is encoded in a floor field or potential. The simulations are performed using JuPedSim [29]. The model is a variation of the collision-free speed model introduced by Tordeux *et al.* [23] without exponential repulsive interactions between the agents. The velocity vector **v**_*i*_ of agent *i* is determined by

3.1 $$v_{i}=V(s_{i}(x_{i},x_{j},\ldots ))\cdot e_{i},$$

with **e**_*i*_ the movement direction and

3.2 $$V(s_{i})=\min\{v_{0},\max\{0,(s_{i}-l)/T\}\}.$$

The speed of agent *i* is therefore determined by the desired speed *v*_0_, the diameter of the agent *l*, the minimal spacing *s*_*i*_ and the slope factor *T*. The minimal spacing is given by

3.3 $$s_{i}=\min_{\;j\in J_{i}}s_{i,j},$$

where *J*_*i*_ is the set of agents in the headway of agent *i*,

3.4 $$J_{i}=\left\{\;j,e_{i}\cdot e_{i,j}\leq 0\wedge |e_{i}^{⊥}\cdot e_{i,j}|<\frac{l}{s_{i,j}}\right\}.$$

The vector $e_{i}^{⊥}$ is perpendicular to **e**_*i*_. The set *J*_*i*_ is illustrated in figure 3*a*.

The desired direction **e**_0_ is determined by a floor field [22]. In this case, the continuous floor field consists of two parts, which are implemented into JuPedSim. To find the quickest way to the exit, the ‘Eikonal equation’ [30]

3.5 $$|\nabla c(x)|=F(x),\;x\in \Omega ,\;c{|}_{\delta \Omega }=0\;$$

is solved on the spatial domain Ω, where δΩ marks the target domain. The slowness field *F*(**x**) determines the speed of a particle in the floor field *v* = 1/*F*(**x**), while *c*(**x**) is the time-cost to the target domain. The interactions with the wall are built into the floor field by a slowness field near the walls. The slowness is linearly decreasing with the distance to the wall, with a minimal value of 0. This point is the wall-avoidance distance *d*_*w*_ set to 0.25 m in the simulations. For more details, see [29,31]. An example of a floor field is represented in figure 3*d*. The resolution for the floor field used in the simulations is Δ*h* = 0.01 m. Because of the collision-free property of the model [23], agents can get into a deadlock, setting their speed to 0. To solve this problem, a white noise term is added to the desired direction which also captures the imperfect choice of direction:

3.6 $$e_{i}=\frac{e_{0}+\zeta }{Z}.$$

**e**_0_ is the direction indicated by the floor field (i.e. the desired direction of the motion) and **ζ** a random direction vector. Both components of **ζ** are determined by a normal distribution with zero mean and variance *σ*. The vector is normalized by *Z*.

### 3.1. Model parameters and psychological factors

The parameters of the agent-based model are strongly connected with fundamental diagrams, which are empirical relations between flow, density and speed. A special representation of the fundamental diagram is the speed distance function equation (3.2), including the model parameters *v*_0_ the desired speed, *T* the slope factor and *l* the minimal distance between agents. Experimental studies on the fundamental diagram have shown that they depend on a set of individual properties *P* = {*P*_1_, *P*_2_, …} of pedestrians like culture, body height, gender, or motivation [32–37]. However, they also depend on various environmental factors *E* = {*E*_1_, *E*_2_, …} like the width of the facility, the presence of rhythm or background music [38,39] or visibility [40]. In the model, this would translate to a dependence of the parameters on these factors, e.g. *T*_*i*_ = *f*_*i*_(*P*, *E*) and *v*_*i*,0_ = *g*_*i*_(*P*, *E*) for the agent *i*. Thus, the parameters of the agent-based model describe environmental, physical as well as psychological properties of pedestrians.

The current state of research does not allow identifying a set of major factors or even to estimate a functional form. Moreover, in general, it is challenging to disentangle the influences of these factors. Adding to this complexity, the parameters are also connected to a stimulus–response mechanism of perceiving the environment and properties of the musculoskeletal system, leading, for example, to the movement of a pedestrian by steps. Due to this complexity, many simplifications have been made for a viable theoretical description.

### 3.2. Parameters and initial conditions

The desired speed is fixed at *v*_0_ = 1.34 m s^−1^ which is the empirically estimated mean walking speed for unimpeded pedestrians [41]. The other free model parameters are estimated using experimental data [4]. Agents are represented by non-deformable discs with fixed diameter *l*. The exclusion property of the model then yields an upper bound for the density. To estimate the agent diameter from the experimental data, the maximum density *ρ*_max_ which is slightly above 9 m^−2^ during high motivation runs is taken as a pivot (figure 2).

Assuming that this maximum density corresponds to a (hexagonal) dense packing of discs, one finds that *r*_*a*_ = 0.175 m corresponds to a maximal density of *ρ* ≈ 9.4 m^−2^. Simulations with this value yield good results. See section ‘Material and methods’ for more details.

#### 3.2.1. Motivation and slope factor *T*

As discussed above, certain simplifications have to be made for a viable description. We assume that the parameters do not depend on the agents, e.g. *T*_*i*_ = *T* for all *i*, and do not change during an experimental run. The first assumption is reasonable as experiments are usually performed with a relatively homogeneous group of participants (e.g. students). The effects of the different levels of motivation studied in the experiments [4] are modelled by different values of *T*. The idea behind using *T* as the parameter to model the effect of motivation is that with highly motivated and jostling pedestrians, any gap between neighbours will be filled immediately, corresponding to a smaller value of *T*. Pedestrians moving with low motivation keep distance in dependence of the walking speed, which corresponds to a larger value for *T*. Figure 3*c* depicts the speed function (3.2) for different values of *T*. The slope factor *T* determines at which distance agent *i* reacts to the closest neighbouring agent in its path (i.e. a neighbouring agent *j* that satisfies (3.3)) and the change of speed in dependence of the distance between pedestrians *i* and *j*. In the limit *T* → 0, the speed function reduces to simple volume exclusion, where an agent moves with its desired speed even if the distance to the neighbour is small. In the case of collision with a neighbouring agent, the velocity is instantaneously set to 0.

The values are chosen to be *T* = 1.3 s for agents with low motivation and *T* = 0.1 s for high motivation, as these values fit well to the data. In the simulation, an agent with low motivation reacts to an agent in its headway when the distance *s*_*i*_ − *l* ⪅ 2 m. For an agent with high motivation, the critical distance to trigger a reaction is *s*_*i*_ − *l* ⪅ 0.5 m (see equations (3.2)–(3.4)).

#### 3.2.2. Initial density and population size

**Figure 4.**
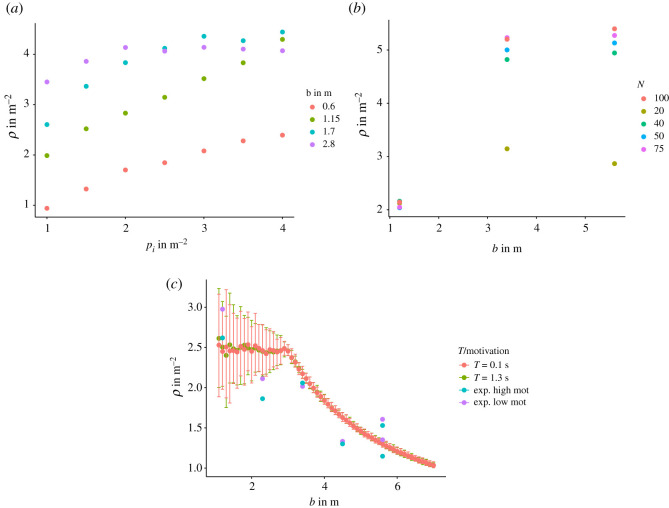
(*a*) Mean Voronoi density after 10 s in respect to the initial density in the corridor for different corridor width *b*. (*b*) Mean Voronoi density after 10 s in respect to the corridor width *b* for a different number of agents *N*. (*c*) Mean initial Voronoi densities *ρ_i_* in respect to the corridor width. The initial density is calculated for the whole corridor from *y*_min_ = 0 m to *y*_max_ = 7.0 m. Comparison of experimental and simulation densities for high and low motivation.

Next, we analyse the influence of the initial density *ρ*_*i*_ on the mean density *ρ* in the measurement area during the measurement time. Figure 4*a* depicts the mean value of the density between 10 s and 15 s with respect to the initial density for different corridor widths. The initial density has a non-negligible influence on the density for the measurement interval. This influence is especially pronounced for narrow corridors (*b* < 3.4 m) and weakens for wide corridors (*b* > 3.4 m) as *ρ*_*i*_ > 2 m^−2^. The initial Voronoi densities *ρ*_*i*_ in the experiment are roughly identical and can be found between 1.5 and 3.0 m^−2^ (figure 4*c*). To meet this criterion, the corridor length *l*_*c*_ is adapted so that the agents can be evenly distributed over the area of the corridor and meet the initial conditions. The length is set to *l*_*c*_ = *N*/*bρ*_*i*_ up until the corridor length would be smaller than 7 m (the corridor length in the experiment). From there, the corridor length is set constant at 7 m. The initial density *ρ*_*i*_ is chosen randomly from a uniform distribution U(2.0,3.0). Figure 4*c* shows the experimental initial densities compared to the simulation. For corridors with *b* < 3.4 m the corridor length *l*_*c*_ < 7 m and have therefore a larger range of values. As already discussed, runs from the experiment with fewer than 42 participants are excluded.

Figure 4*b* illustrates that in the model for *N* > 40 the number of agents has a limited but not negligible influence on the mean density. To obtain comparable results for the mean Voronoi density with respect to the corridor width, the number of agents in the simulation is set constant to *N* = 55 in the middle of the range of participants. The simulation runs are calculated in a range of *b* = [0.8, 7.0] m in 0.1 m steps. For every value of *b*, 500 simulation runs with random initial conditions are conducted. This makes 31 000 for each motivation *T* = {0.1, 1.3} s and 62 000 simulations in total for the main analysis. The model parameters are summarized in table 2.

**Table 2.** Summary of model parameters and values used in the simulations.

parameter	variable	value
slope factor (motivation)	*T*	{0.1, 1.3} s
desired velocity	*v*_0_	1.34 m s^−1^
agent size (hardcore exclusion)	*l*	0.35 m
noise variance	*σ*	0.7
population	*N*	55
floor field resolution	Δ*h*	0.01 m
wall avoidance distance	*d*_*w*_	0.25 m
corridor width	*b*	[0.8, 7.0] m

## 4. Results

**Figure 5.**
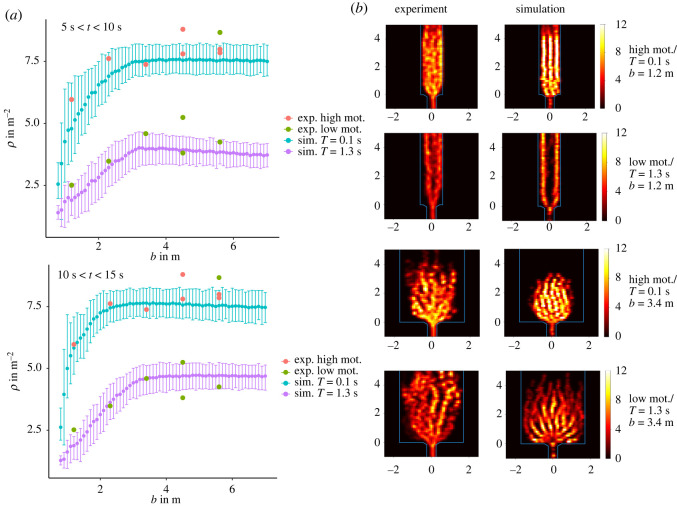
(*a*) Top: mean density in the measurement area calculated with the Voronoi method from 5 to 10 s for different values of the slope factor *T* and motivations in the experimental data. The simulation runs were conducted 500 times for each corridor width *b*. The error bars show where 95 % range of the mean. Bottom: the same as above from 10 to 15 s. (*b*) Density fields for different motivations and corridor width *b*. Left column depicts the experimental data, while the right column shows the simulation results. From top to bottom, the motivation is alternating between high motivation (*T* = 0.1 s) and low motivation (*T* = 1.3 s). The corridor width is *b* = 1.2 m in the top panels and *b* = 3.4 m in the bottom panels.

In the experimental data, the measurement interval for the mean density was chosen to be between 5 s and 10 s. This interval was chosen to have comparable results as the participants’ number varies between the runs and, as discussed above, influences the mean density. Since the simulations are controlled for the agent number, it is also interesting to look at a second interval and compare it to the experimental data. Here, the interval from 10 s to 15 s is chosen. Figure 5*a* shows the simulation results and the experimental data. The error bars show the 95% interval of the 500 runs conducted. The simulations reproduce the increase in density with the corridor width *b* without the adjustment of any other parameter for a given motivation. The increase in density from low to high motivation can be reproduced by only adjusting the slope factor *T*. The mean density measured between 10 and 15 s shows a monotonic increasing behaviour until it reaches a saturation point at about *b* = 3.2 m. For low motivation (*T* = 1.3 s), the density is non-monotonic. The experimental data lie with 19/28 points mostly inside the 95% interval, while the points outside do so by a small margin. Especially, the runs for *b* < 4.5 m are in good concordance with the experimental data. The runs for *b* = 4.5 m show the largest deviation from the simulation results for high motivation. As discussed in the section ‘Experimental data’, the high motivation experimental runs for *b* = 4.5 m do not reach a steady state. In the low motivation data, there is an outlier for *b* = 5.6 m where the density is comparable to that of the high motivation scenario. This is the largest run with *N* = 75 participants. Figure 4*b* shows the density for low motivation and a different number of participants, but the increase in the number of agents cannot explain the high value of the measured density.

To improve the understanding of the overall dynamics in the corridor, the density field of a single run is calculated according to equation (5.3). Figure 5*b* shows the density fields for *b* = 1.2 m and *b* = 3.4 m from the experiments and simulations. For low motivation and *b* = 1.2 m, two equally dense lanes form in both the experiments and the simulations, which merge near the exit. With high motivation, three lanes form in the simulation and the overall density increases. In the experimental case, the overall density increases, but it is not possible to identify clear lanes. For low motivation with *b* = 3.4 m, clear lane formation is also evident in both the experiments and the simulation. A noticeable difference is that while the agents in the simulation are broadly distributed over the whole corridor near the *y* = 0 line, the distribution of participants is wedge-shaped near the exit. One potential reason for this are the barricades in the experiment, which have metal gratings in front of them (figure 9*c*). These seem to be mostly avoided by the participants of the experiment, especially in the low motivation runs, and therefore could act as an obstacle. This becomes clearer when looking at the videos of the experiments, which can be accessed at [42]. In the simulations, this circumstance was not taken into account in order to keep the geometry simple as the wedge shape reduces or disappears altogether for the case of high motivation (figure 9*d*). In the high motivation case, the participants in the experiment and the agents in the simulation are more compressed than in the low motivation case. For both motivations, distinct lanes can be observed. The wedge shape in the density profile is still noticeable, but less pronounced in the experimental data.

**Figure 6.**
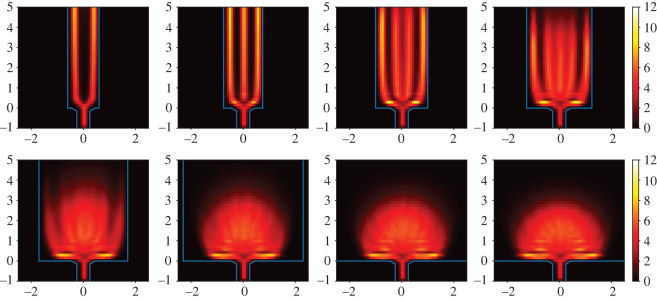
Mean density fields (m^−2^) on the *x*–*y* plane (m) for all 500 runs from 10 to 30 s for *T* = 1.3 s. The top row shows the runs with corridor width *b* = {1.2, 1.5, 2.0, 2.5} m. The bottom row shows values for *b* = {3.4, 4.6, 5.8, 7.0} m.

**Figure 7.**
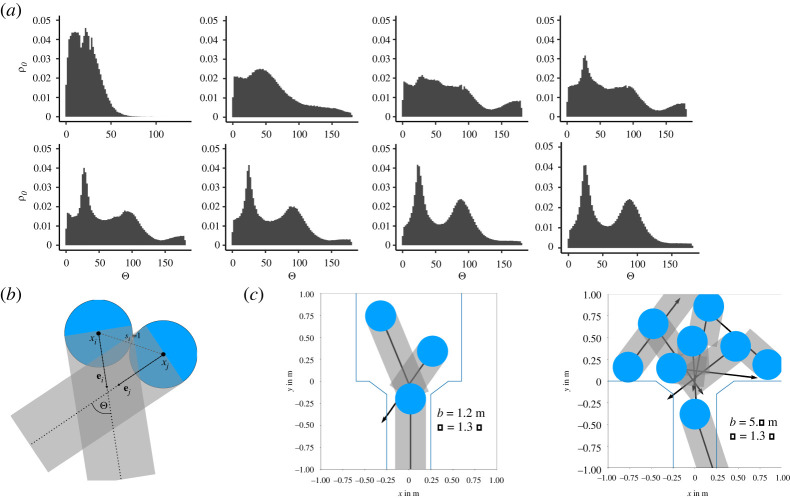
(*a*) Interaction angle distribution for all 500 simulations from 10 s for *T* = 1.3 s. The top row shows the runs with corridor width *b* = {0.9, 1.4, 1.7, 1.8} m. The bottom row shows values for *b* = {2.0, 2.3, 3.0, 4.5} m. (*b*) Illustration of a deadlock situation and the interaction angle. (*c*) Configuration in front of the bottleneck from a simulation with *b* = 1.2 m and *b* = 5.6 m.

A clearer picture of the dynamics in the region of increasing density compared to the saturated region is obtained in figure 6, where the mean density field of selected corridor widths is depicted of all 500 runs. The top row shows runs with *b* < 3.2 m, while the bottom row depicts runs with *b* > 3.2 m. It is visible how the widening of the corridor has a major influence on the dynamics of the evacuation process. As *b* increases, new lanes form and the interaction angle at the merger point between the direction of the agents and the direction to the exit steepens. This increases the potential for conflicts near the exit and thus the density. For *b* > 3.2 m, the influence of the corridor width is small as the formation of new lanes has little influence on the situation near the bottleneck. Electronic supplementary material, figure S2, shows an analogous comparison for high motivation simulations presenting a similar behaviour. Figure 7*a* depicts the distribution of the interaction angle, as defined in figure 7*b*, between two agents for varying *b* in a radius of *r* = 1 m of the point **r** = **0**. In this region, the merging of lanes takes place (figure 6). The interaction angle is defined as the angle between the noiseless desired direction of one agent and the agent they are interacting with (i.e. the agent which fulfils the conditions according to equations (3.3) and (3.4)). Noiseless means that at every step of the simulation with noise acting on the dynamics according to (3.6) the interactions are calculated according to the Eikonal equation (3.5) without the addition of noise. This equates to the expected interaction angle for the specific configuration. The transition of the distribution exhibits an interesting behaviour where peaks existing at smaller *b* disappear as *b* widens and new peaks emerge until the distribution stabilizes with two distinct peaks. In general, the likelihood of larger interaction angles increases with *b*. The shape of the distribution can be divided into three regions. When *b* is small (*b* < 1.4 m) the likelihood for the interaction angle is highest for small angles $\Theta <50^{\circ }$ and monotonically decreasing for larger angles. When *b* > 1.4 m and *b* < 1.8 m the distribution widens with a higher likelihood for large interaction angles up to 180°. For *b* = 1.7 m, the interaction angle distribution is rather flat in comparison with three less distinct peaks at 30°, 50° and 100°. As *b* increases to 1.8 m, a new peak emerges at around 30° turning the distribution uni-modal. Furthermore, increasing *b* turns the distribution bi-modal with a narrow peak around $\Theta =30^{\circ }$ and a wider peak at $\Theta =90^{\circ }$. This structure is stable for large *b* > 3 m. Interactions at larger angles have a higher potential for conflicts because the noise term is not as sufficient in solving these conflicts as in the case of smaller angles where pedestrians follow each other. For high motivation (*T* = 0.1 s; electronic supplementary material, figure S4) the behaviour is similar, but the transitions take place at smaller *b*. The distribution converges into a bi-modal form, but with a more pronounced peak for smaller angles and a smaller peak for larger angles.

Another observable measured in the experiments of Adrian *et al.* [4] and Garcimartin *et al*. [9] is the waiting time *T*_*w*_ of the pedestrians as a function of the distance to the door *r* at time *t* of the run. In the experiments of Garcimartín *et al.* [9], the participants are not restricted by a corridor, i.e. the corridor can be assumed as infinitely wide. As argued in the study, it is expected that the evacuation time follows a power law distribution $T_{w}\propto r^{\alpha }$ with *α* = 2 in the case of a wide corridor. The reasoning behind this is that the evacuation time for a single pedestrian leaving the area should scale linearly with *r*. In a crowded evacuation scenario, pedestrians interact with each other. Assuming that the pedestrian motion can be approximated as a fluid with laminar flow implies that the speed scales with 1/*r* because of the continuity condition. Therefore, the evacuation time scales with *r*^2^. For single-file motion in a narrow corridor, the evacuation time should scale linearly as the velocity is independent of the distance to the door and therefore *α* = 1.

**Figure 8.**
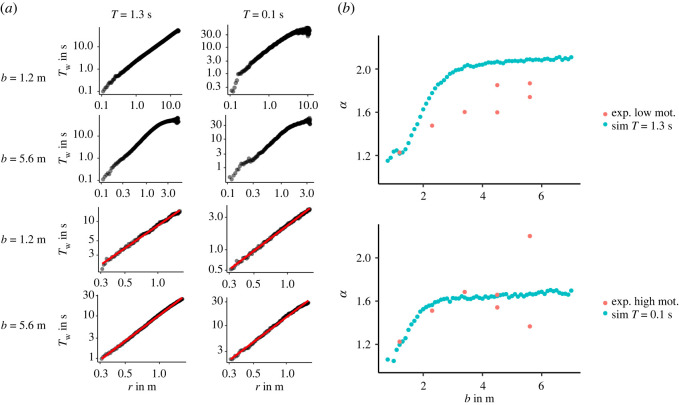
(*a*) Waiting time to target *T*_*w*_ in respect to the distance *r* from the target between 10 and 20 s with all 500 runs pooled together. The left column depicts runs with *T* = 1.3 s while the right column depicts runs with *T* = 0.1 s. The alternating order from top to bottom shows the results for *b* = {1.2, 5.6} m for different cutoff distances of the data (no cutoff for top two values and 1.5 m for bottom). (*b*) Exponent of the power law for the waiting times for low motivation and *T* = 1.3 s in the top panel and high motivation and *T* = 0.1 s in the bottom panel.

**Figure 9.**
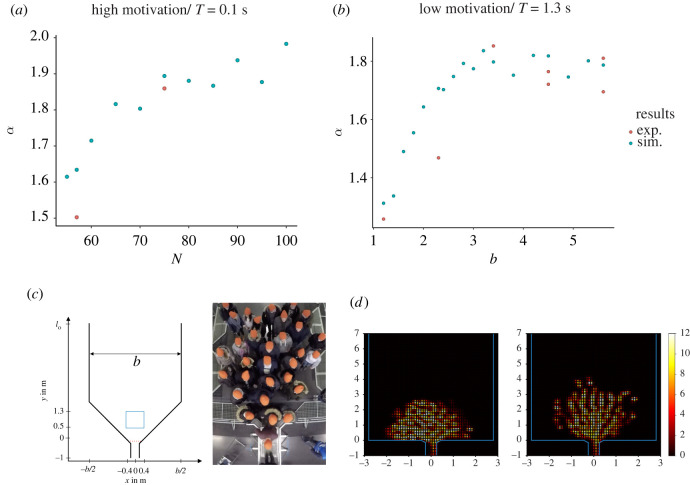
(*a*) Simulation results for *α* in respect to the number of agents *N* for *b* = 5.6 m between 10 and 20 s. Every data point is the mean of 50 simulations. (*b*) Exponents *α* of the power law for the waiting times for low motivation using the hopper-shaped geometry for *T* = 1.3 s shown in (*c*). (*c*) Illustration of the geometry with hopper-shaped walls and a snapshot from an experimental run [4] with low motivation. (*d*) Heat maps for the experimental runs with high motivation (left) and low motivation (right) with a corridor width of *b* = 4.5 m.

To test this relation in the simulation, the evacuation time is measured for all runs between 10 s and 20 s. In this time interval, the distance to the exit is measured along a straight line from the point **r** = **0**. Simultaneously *T*_*w*_ is calculated for every agent. This is done in 0.5 s intervals so that the agents’ position varies. Figure 8*a* shows the log–log plot of *T*_*w*_ as function of *r*. The top four panels show the evacuation time for all distances. The power-law relation is valid for a distance from *r* = 0.3 m up to around *r* = 1.6 m. Figure 8*b* shows the exponent *α* of *T*_*w*_ in respect to the corridor width *b* for both motivations. For the smallest *b*, the exponent is close to one. With low motivation *α* increases and saturates as expected around *α* = 2. The comparison with the experimental data from Adrian *et al*. [4] shows a close match to the simulation data only for *b* = 1.2 m. In the high motivational case, the exponent converges to a value of about *α* = 1.7 and shows a closer concordance to the experimental data. The values for *b* = 5.6 m exhibit a large spread, a phenomenon that is attributed to the different number of participants of each test (the smaller value was obtained for *N* = 57 participants while the larger one for *N* = 75). Figure 9*a* shows *α* for simulations with different numbers of agents for *b* = 5.6 m and *T* = 0.1 s. The number of agents influences *α*, which goes to *α* = 2 for *N* ≈ 100. The smaller exponent may stem from the fact that due to the compact cluster of pedestrians a full semicircle is not present in the region analysed, leading to a lower exponent (see electronic supplementary material, figure S2). In general, the high motivation runs fluctuate stronger than those for low motivation. The deviation of the simulation from the low motivational empirical data could be a result of the wedge-shaped configuration of the participants discussed earlier, as this influences the geometry in front of the bottleneck. To verify these, simulations with different geometries are conducted. Instead of a horizontal line for the lower walls as illustrated in figure 3*b*, the wall is continued from the exit with a 45° angle until the intersection with the vertical wall (figure 9*c*). The results for different *b* are depicted in figure 9*b*. The simulations were repeated 50 times. The value for *α* saturates around *α* = 1.8 and is in closer concordance with the experimental results.

**Figure 10.**
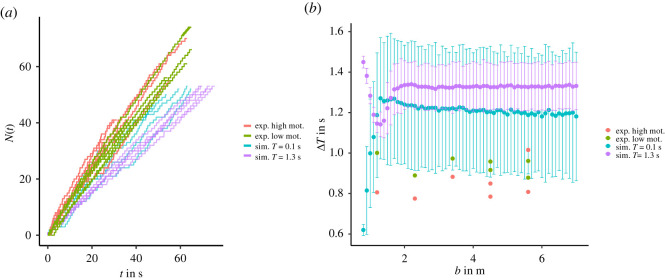
(*a*) *N*–*t* diagram for the experimental data (vibrant colours) with high motivation (red) and low motivation (green). Analogous exemplary simulation data (pale colours) for the same corridor width *b* as in the experiments, using one run per width. (*b*) Simulation and experimental results for the time gap Δ*T*. The error bars show the 95% interval of the simulation data for 500 runs.

Figure 10*a* depicts the *N*–*t* diagram and figure 10*b* the mean of the time gap Δ*T* between consecutive agents crossing the *y* = 0 m line taken over all 500 simulations, excluding the first and last 10 agents of each run, analogous to [4]. The time gap is proportional to the inverse of the flow Δ*T* ∝ *J*^−1^. The comparison of the results shows that in the model, the agents are less efficient in reaching their target compared to the experimental data. The mean time gaps increase from small *b* but plateau quickly at around *b* = 1.8 m. This kind of behaviour is not found in the experiments. The faster-is-slower effect [43] cannot be observed, in both the model and the experiments. This is in agreement with experiments by Haghani *et al.* [10] where aggressive pushing was prohibited and also with recent numerical studies using the social force model [26]. The margin for the data points of different runs is wider for *T* = 0.1 s compared to the runs with large *T*. This is due to the increased occurrence of clogs in the system, as can be observed in the *N*–*t* diagram (figure 10*a*). In the experimental data, a similar behaviour can be observed where the initial slope of the curve is larger, but due to clogs the flow can be inhibited for longer times. More data are needed to make a more conclusive statement, but a higher probability for larger Δ*T* is reported in [44]. In the model, the agents can only react to the presence of other agents in their path, but are not able to communicate their intentions to their surroundings or anticipate the behaviour of other agents. This could point to the missing of a cooperation and negotiation mechanism [1] in the model, supported by the increase of Δ*T* with increasing *b*, as conflicts get more prevalent.

## 5. Discussion and conclusion

This study sheds new light on the question of psychological influences on pedestrian dynamics in bottleneck flow with varying boundary conditions discussed in previous experimental studies [3,4]. A model with simple agent interactions can reproduce the empirical results [4] surprisingly well using a single parameter to model different motivational states. In the simulations, all parameters are static and homogeneous for a specific run and only the boundary condition *b* and the motivation, with effects on the behaviour of the agents modelled by the time gap parameter *T*, are varied in the initial conditions. The results for the density in front of the bottleneck, the relationship between time to target *T*_*w*_ in respect to the distance *r* and the formation of lanes in narrow corridors correspond well to the empirical findings. The simplicity of the interactions in the model demonstrates that, on the one hand, there is no obvious indication for the relevance of social psychology to explain the increase of the density with the width of the corridor and the lane formation in narrow corridors. On the other hand, motivation and social norms may be relevant to describe the changes in the density with varying motivation levels. In particular, the experimental observation stating that, for very wide corridors, low and high motivation lead to the same density level (see figure 5*a*, *b* = 5, 6 m and *ρ* ≈ 7, 8 m^−2^) is not explainable by the approach presented here. Summarizing, the results illustrate the challenge in distinguishing possible effects of social psychology on the crowd behaviour from behaviour emerging from self-organization through microscopic interactions in a physical sense, even in rather simple scenarios.

### 5.1. Material and methods

The code used for the simulations can be found at [45] and the raw data of the pedestrian trajectories are in a repository [46]. The main focus of this work is on the mean density in front of a bottleneck. Two different measures for the density are used. To be consistent with [4], the mean density in the measurement area is measured using the Voronoi method introduced in [47] since it produces smooth density curves. The Voronoi density is defined using a Voronoi diagram. In the case of a two-dimensional Euclidean space **X**, the space is divided into subregions called Voronoi cells. The positions of the *N* agents **r**_*i*_, *i* ∈ {1, *N*} at a given time act as vertices of the Voronoi diagram. A point in space belongs to the Voronoi cell *R*_*i*_ of agent *i* if the Euclidean distance *d*(**r**_*i*_, **x**) to agent *i* is minimal compared to all other agents. *R*_*k*_ is the set

5.1 $$R_{k}=\{x\in X|d(x,r_{i})\leq d(x,r_{j})\;\forall \;j\neq i\}.$$

The mean Voronoi density in an area *A* is then defined as

5.2 $$\rho_{0}=\frac{\int_{A}p(x)\;dx}{\left|A\right|},$$

where $p(x)=\sum_{i}p_{i}(x)$ and *p*_*i*_(*x*) = 1/*A* if **x** ∈ *A* and 0 otherwise.

A second approach to calculate the density field over the whole corridor area is used to get detailed density maps, with more clearly defined boundaries of the agents. In this case, the local density *ρ* in the system can be defined as

5.3 $$\rho (r;X)=\sum_{i=1}^{N}\delta (r_{i}-r),\;\rho (r)=\langle \rho (r;X)\rangle ,$$

where **r** is the position, **X** marks a configuration and *δ*(*x*) denotes the Dirac delta-function. The mean 〈〉 is taken over the configurations **X**. Since the resolution in the numerical simulations is finite, the delta-function is approximated by a Gaussian:

5.4 $$\delta (x)=\frac{1}{\sqrt{\pi }a}\exp\left[-\frac{x^{2}}{a^{2}}\right].$$

Density and flow are measured in the same way as in the experimental set-up described in the previous section, using the Voronoi density (5.2).

### 5.2. Estimation of agent diameter

**Figure 11.**
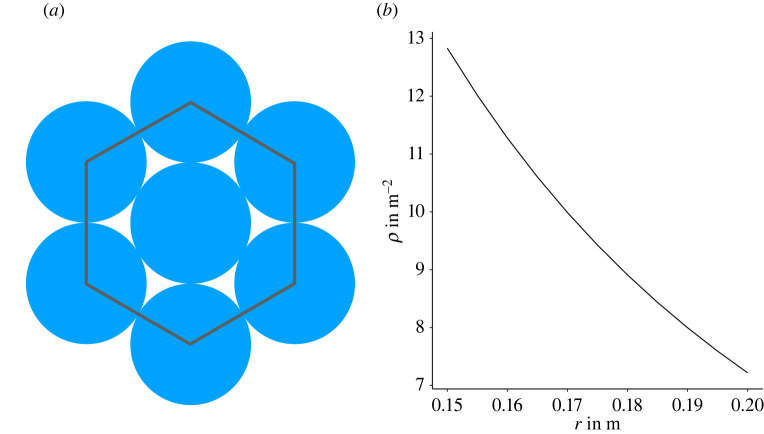
(*a*) Circles in hexagonal packing. The area inside the grey hexagon is used to determine the packing density in (5.5). (*b*) The hexagonal packing density in respect to the radius *r* of a disc.

In two dimensions, the maximal packing fraction that can be achieved (by a hexagonal packing) of identical discs with radius *r* = *l*/2 is $\eta =\pi /\sqrt{12}\approx 0.9069$ (figure 11*a*). This provides an estimate for the upper bound of the density:

5.5 $$\rho_{\max}=\frac{\eta }{r^{2}\pi }.$$

Figure 11*b* illustrates equation (5.5). The maximal experimental density *ρ* is slightly above 9 m^−2^ (figure 2). For the simulations, a value of *r*_*a*_ = 0.175 m is chosen, which corresponds to a maximal density of about 9.4 m^−2^, as this yields good results. The agent size chosen corresponds to a rather small shoulder width, since e.g. [48] gives an approximate adult shoulder width of *l* = 0.46 m. However, the circle diameter of 0.35 m could be seen as a contracted pedestrian.

## Data Availability

Data and relevant code for this research work are stored in GitHub: https://github.com/JonasRzez/jpsnewnoise.git and have been archived within the Zenodo repository: https://doi.org/10.5281/zenodo.6458163. The data are provided in electronic supplementary material [49].
